# Learning-by-Concordance Approach in Health Professions Education: A Scoping Review

**DOI:** 10.5334/pme.1658

**Published:** 2025-07-04

**Authors:** Antoine Roche, Ann Alexandra Rodriguez Turcot, Andréanne St-Pierre, Sarah Cherrier, Marie-Claude Audétat, Bernard Charlin, Joseph-Omer Dyer

**Affiliations:** 1 Institut de Formation en Masso-Kinésithérapie (IFMK) de Montpellier, Montpellier, France; 2 UDREM Unit of development and research in medical education, Faculty of Medicine, University of Geneva, Geneva, Switzerland; 3 School of rehabilitation, Faculty of Medicine, Université de Montreal, Quebec, Canada; 4 Faculty of Medicine, University of Montreal, Quebec, Canada; 5 Interdisciplinary research group on cognition and professional reasoning, Center for Research in Health Pedagogy, Faculty of Medicine, Université de Montreal, C.P. 6128 Succursale Centre-Ville, Montreal, H3C 3J7, Quebec, Canada

## Abstract

**Introduction:**

Learning by concordance (LbC) is an educational approach designed to develop expertise, particularly in the field of clinical reasoning (CR) among healthcare professionals. It is based on the script concordance test (SCT) with the addition of feedback based on expert responses. The objective of this study was to map the scientific literature on this rapidly growing learning approach.

**Methods:**

A scoping review was conducted following the Arksey and O’Malley framework and the PRISMA-ScR guidelines. A systematic search was conducted in MEDLINE, Embase, CINAHL, Web of Science and Google Scholar up to March 10, 2025. Eligible primary studies had to focus on the LbC approach targeting healthcare learners.

**Results:**

Twenty-eight studies met the inclusion criteria: twenty focused on the implementation of the LbC approach and eight on its development. Most of the studies used mixed methods (quantitative and qualitative). The results mainly indicate that learners perceive the LbC approach as engaging and beneficial for decision-making. The articles mention five elements related to the development of LbC that would contribute to its success.

**Discussion:**

The LbC approach could be applicable to a wide range of disciplines and learning levels. The variability in the procedures for developing the approach, as well as the variability of the objectives and methodologies of the studies, limit the comparability of the results.

**Conclusion:**

LbC is a promising approach for promoting decision-making skills in a variety of uncertain clinical contexts. The concept of standardized development and evaluation frameworks for this approach could improve its applicability, effectiveness and reproducibility.

## Introduction

Clinical expertise in healthcare professions is intrinsically linked to the ability to make informed decisions in contexts of uncertainty [1,2,3,4,5]. Indeed, healthcare professionals must make decisions to solve clinical problems that are often ill-structured [6,7,8], i.e. problems for which there is uncertainty as to how to solve them [9,10]. Despite its central role in clinical decision-making, the most effective educational strategies for training healthcare professionals in the management of uncertainty remain unclear [11,12,13,14]. Over the past decades, research in health professions education has sought to optimize the training of clinical reasoning (CR) by integrating strategies that improve decision-making skills amid uncertainty [15,16,17,18]. Training in CR must incorporate strategies that explicitly address uncertainty, as clinical problems often do not have a single correct solution and can be resolved by several valid approaches [19,20,21,22,23]. It is essential to develop training approaches to foster the ability to make decisions in contexts of uncertainty, as this skill lies at the heart of clinical expertise.

Script Concordance Test (SCT) is a CR assessment tool that considers the individual’s ability to make choices and decisions in uncertain situations [24]. It has been widely studied in several healthcare fields [25,26,27,28,29]. The development of the SCT is based on the script theory, which postulates that healthcare professionals organize, store and access their clinical knowledge notably through “illness scripts”. These scripts, which develop with clinical experience, enable healthcare professionals to recognize patterns in clinical data that are associated with health conditions, and to make decisions about patient care [25,26,30,31]. The principle of SCT is to evaluate these scripts by presenting vignettes to learners with situations involving uncertainty, to which new data are added for consideration in answering Likert-type multiple-choice questions on the level of plausibility or relevance of a hypothesis or proposed action [32]. To quantify the performance of respondents, SCT weights the responses according to their degree of concordance with the answers of a reference panel, which is usually made up of people experienced in the field under study. In this respect, a learner receives one point for a concordance question when his or her answer matches that of the majority of panelists, a fraction of a point for an answer that matches that of a minority of the panelists, or zero points when no panelist gave that answer [33].

Learning-by-concordance (LbC) is a learning approach directly derived from SCT, since it has the same format, except that it presents respondents with the panelists’ answers and explanations on the concordance questions, with the aim of providing learners with elaborate formative feedback [34]. Borrowing from SCT, LbC training gives learners the opportunity to practice making decisions on diagnostic or action hypotheses in a context of uncertainty, and to receive feedback enabling them to compare their decision-making with that of a reference panel [34]. This innovative learning approach is increasingly used, albeit heterogeneously, in different domains of the healthcare professions [35,36,37]. In 2021, guidelines were proposed to support the design of training courses based on the LbC approach [34]. However, there is currently no comprehensive review on the empirical application and evaluation of the LbC approach in health professions education. Moreover, the evidence for learning and the elements that promote meaningful learning with this approach are unclear.

A scoping review is a rigorous and appropriate method for exploring an emerging field of research and mapping the available literature on a given topic (Arksey & O’Malley, 2005; Tricco et al., 2018). Unlike a systematic review, which aims to assess the effectiveness of an intervention, a scoping review examines the diversity of conceptual frameworks and methodological approaches used in existing literature. This approach is therefore particularly suitable for exploring how LbC is studied and applied in the training of healthcare professionals. It allows for the identification of key determinants that influence the adoption of LbC and highlights the methodological and conceptual gaps that need to be explored in greater depth in future research.

## Objectives

LbC training is an approach that is becoming increasingly important in the literature on strategies for developing expertise in the healthcare professions. This study aims to fill this gap by conducting a scoping review of existing research on LbC, mapping its scope and nature, identifying the methodological frameworks used, and pinpointing gaps in research design and theoretical foundations to inform future research.

## Methods

### Methodological framework

This scoping review was conducted in accordance with the methodological framework established by Arksey and O’Malley [38]. It also follows the Preferred Reporting Items for Systematic Reviews and Meta-Analyses extension for Scoping Reviews (PRISMA-ScR) guidelines [39]. These guidelines ensure the rigor and transparency needed to map the existing literature on a given topic, enabling in-depth understanding while identifying research gaps [40,41,42].

### Identifying the research questions

Based on the objectives of this review, the research team formulated the following research question to guide the literature search: What is the scope and nature of scientific studies on the LbC approach in health professions education? More specifically, this review seeks to identify research questions and methodological approaches used to study the LbC approach, as well as to highlight key gaps in the existing literature.

### Identifying relevant studies

A comprehensive bibliographic search was conducted in MEDLINE ALL (Ovid), Embase (Ovid), CINAHL, Web of Science Core Collection, and Google Scholar, covering all available records from the date of creation of the databases until March 10, 2025. The search strategy was developed in collaboration with a health science librarian (SC) (see Appendix 1). The search strategies included controlled vocabulary (e.g., MeSH terms) and keywords related to three core concepts: “learning-by-concordance”, “clinical reasoning”, and “health professions education”.

Inclusion criteria were as follows: studies had to (1) be original and present primary data collection; (2) be published in a peer-reviewed journal; (3) use LbC as the main educational intervention; (4) target learners who are health science students or healthcare professionals; (5) aim to teach CR or any other form of expertise; and (6) be published in English or French.

Exclusion criteria were as follows: (1) conference abstracts; (2) non-original studies, i.e., based on secondary analyses such as literature reviews; and (3) studies primarily focused on SCT and not using the LbC approach (i.e., using panelists feedback as a training method). These criteria are consistent with recommendations for scoping reviews, and the objectives of this study, which are to extract validated data presented in a structured manner and to reduce potential biases associated with unpublished or non-peer-reviewed sources [38,42].

### Study selection

Data extracted from bibliographic databases were exported to the Covidence bibliographic sorting platform (https://www.covidence.org/). Studies were selected using a two-stage iterative approach: 1. Screening: After Covidence platform had eliminated duplicates, the titles and abstracts of the identified articles were independently reviewed by two reviewers (AAR and ASP) to determine their eligibility. A third reviewer was responsible for ruling if disagreement remained after discussion (AR).

2. Eligibility: The full texts of potentially relevant articles were evaluated for inclusion by two independent reviewers (AAR and ASP). Disagreements were resolved by discussion. A third reviewer was responsible for ruling if disagreement remained after discussion (AR).

### Charting the data

Data from the selected studies were extracted and classified in a table (Excel, Microsoft) with pre-determined variables. The data extracted for each study included: authors, year of publication, type of study, objective, methodological design, learner population, type of intervention, creation process, comparator, learning domain, description of LbC educational intervention, main study results, and conclusions. Data entry was carried out by three independent reviewers (AAR, ASP, AR), who then cross-checked the extracted data.

### Collating, summarizing and reporting the results

The data extracted from the studies were systematically analyzed to produce a synthesis. First, a descriptive approach was used to categorize the characteristics of the selected studies, including research design, the methodological approaches used to examine LbC, the learner populations involved, how LbC was implemented, and the context of its use. To this end, the analysis followed the PCC framework: Population (P) – learners in health professions education, Concept (C) – Learning-by-Concordance (LbC) considering its description or operationalization as an educational method, and Context (C) – learning contexts such as continuing education, academic and clinical contexts [43] (See Appendix 2).

In addition, a content analysis was conducted to systematically classify and structure the data extracted from the selected studies that are related to the concept of LbC and, more specifically, the important elements to consider for the success of this approach in terms of learning benefits [44,45]. This approach allowed for an objective synthesis of the ways LbC has been implemented and studied in different contexts, with the aim of optimizing the learning of expertise. These analyses and the synthesis of the results were carried out iteratively by two reviewers (AAR, ASP), who consulted each other and worked collaboratively throughout the process. The analyses were cross-checked by a third reviewer (AR) (See Appendix 3 and 4).

### Stakeholder consultation

To enrich our analysis and enhance the relevance of our interpretations, we consulted experts (n = 3) in the field of CR and health professionals education. These experts included authors of publications on CR (n = 2, MCA, BC), and an educator involved in developing training courses for healthcare professionals (JOD). A summary of the preliminary results was sent to these experts for comment. Their comments were incorporated into the interpretation of the results.

## Results

### Sorting of Selected Studies

The search in the databases yielded 596 references. After removing 270 duplicates, 326 references remained. After checking the titles and abstracts, 288 references were excluded. The full-text review led to the exclusion of 10 references, leaving 28 studies that met the criteria (Appendix 4).

### Objectives and methodological characteristics of selected studies

Of the selected studies, 20 focused on the implementation of training courses using the LbC approach and on the impact of the training on the learning of healthcare learners [36,37,46,47,48,49,50,51,52,66,67,68,69,71,72,74,75,76,79,80]. Among these implementation studies, nine adopted a mixed methods design, combining quantitative descriptive analyses (e.g., questionnaire results) and qualitative content analysis of learners’ discourse regarding their opinion on the approach [36,37,47,49,51,52,66,68,80]. Six studies used qualitative approaches, employing content analysis of participants’ discourse to explore learners’ experiences with the LbC approach [46,48,67,69,74,79]. Two studies adopted a mainly quantitative approach: one relied solely on data collection through questionnaires [50], while the other was a quasi-experimental study comparing two cohorts (control vs. intervention) using a customized assessment [76]. Three studies were descriptive implementation reports: two did not collect formal data [71,72], while one reported informal comments from students [75].

In addition to these implementation studies, eight examined the development and conceptualization of LbC training [31,53,54,55,70,73,77,78]. Two of these studies used a mixed methods design, integrating data from questionnaires and a qualitative analysis of participants’ opinion on the training [53,55]. Three studies used mainly qualitative methodologies: One applied a dialogic action research approach with thematic analysis [54]. Another used a nominal group technique to validate an operational framework for the development of LbC [31]. The third followed an interpretive description methodology, conducting four semi-structured dialogue groups [78]. Two studies were descriptive theoretical papers, focusing on the conceptual and pedagogical foundations of LbC [70,73]. One study used a modified e-Delphi method, conducting two rounds of expert consultation to validate a clinical reasoning assessment rubric [77].

### Measuring the effects of the LbC approach

Trends emerge from the results on the potential impacts of the approach [36,37,46,47,48,49,50,51,52,67,68,69,70,71,72,75,76,79,80]. All studies on the implementation of LbC considered learners’ perceptions of LbC, which were mainly assessed through questionnaires, qualitative feedback, or a combination of both. In all these studies, learners consistently appreciated the approach, reporting it as engaging, interactive, and beneficial for the development of expertise. Some studies reported that learners expressed positive perceptions regarding their engagement in LbC, with self-reported improvements in knowledge organization and professional reasoning [36,48,49,51,67,68,71,72,75,76,79,80].

### Population targeted

The “population” category of the PCC framework enabled classification of the information extracted according to the learners targeted. Most studies (60%) examined the use of the LbC approach among medical learners, including medical students [31,37,46,48,49,53,54,66,79] and practicing physicians [36,50,55]. Other studies focused on LbC training among nursing [47,69], speech-language pathology [51,76], dental [52,80], pharmacy [67], and veterinary medicine students [75].

### Concept of the LbC approach

#### Development of the LbC training

The “concept” category of the PCC framework enabled the inclusion of much of the information extracted from the selected studies. Through the selected studies, the LbC concept included the way in which this approach is developed and implemented. The development process appears to be integral to the concept of the LbC approach. The development of LbC training followed distinct methodological steps from one study to another. This process could be based on an assessment of educational needs [47,78] and/or a literature review of relevant theoretical foundations [49,68,76]. Several studies emphasized that the design of vignettes should be aligned with defined learning objectives [51,53,74,80] and that it could be based on established theoretical frameworks [31,52,55,73,77]. The development teams included educators [46,49,51,52,53,54,78], clinicians [31,36,67,69,72,75,79], experienced medical specialists actively involved in postgraduate training and clinical practice [50,68,71], and occasionally educators from other health professions such as pharmacy [67], nursing [69,70,73], dentistry [80], and veterinary medicine [75]. The most frequently cited characteristic for vignette designers was their clinical expertise in the target domain [31,36,37,46,50,52,68,71,72,75,79]. Some studies mentioned collaborations with program directors [47] and faculty members [49,78], who played a key role in aligning the design of the training with the institution’s curricula and educational objectives. One study indicated that vignettes were designed by a single author [51], while others noted that designers received specific training before contributing [50,53,68]. Moreover, some authors have explicitly documented the involvement and significant contribution of students in the development or pilot testing phase to ensure the appropriate level of difficulty and the relevance of the scenarios [68,76,80].

The validation of the vignettes was described as an important process in the design of the training courses based on the LbC approach. Most studies reported a process for validating vignettes and associated questions. This process was usually conducted by experienced clinicians and/or experts in the field [31,46,50,53,54,67,68,69,71,72,75,76,79,80], generally selected on the basis of their clinical experience, recognition from their peers, and their theoretical expertise [48,49,67,68,69,71,72,76,79,80]. Several studies have emphasized the participation of students in the selection of experts or in the pre-testing of vignettes to ensure their adequacy and relevance to their level of training [48,49,68,76]. In addition, some authors have explicitly described the training provided to experts by vignette designers to maintain the consistency of validation criteria [32,47,68].

The number of experts involved in the validation process varied from one study to another, generally ranging from four to twelve per training program [36,47,67,68,71,72,75,76,79,80]. In most studies, experts’ responses were collected using Likert scales with justification boxes for open-ended responses [36,46,47,48,49,50,51,52,67,68,69,71,72,75,76,79,80]. Several studies have adapted the Likert scale structure according to the learners’ levels of experience, including three response levels for novice learners [46,75], five for more experienced learners [36,46], and four in judgment-based LbC training [47,48].

#### Delivery of the LbC training

The way in which the LbC approach is implemented appears to be a central element of the concept underlying this approach. In all the studies, the LbC training format followed a structured approach comprising the following steps: 1) Presentation of a clinical vignette, followed by a diagnostic or intervention hypothesis; 2) Introduction of additional clinical data, requiring learners to assess the plausibility of the hypothesis. 3) Selection of the answer using a Likert-type scale, followed by a justification in an open response field. 4) Comparison with expert responses, with structured feedback.

The structure and content of the feedback varied across studies, reflecting differences in instructional design and intended learning outcomes. The main types of feedback reported included: 1) experts’ justifications for their answers [36,49,67,68,69,71,72,75,76,79,80]; 2) statistical distributions of the expert responses [36,49,67,68,75,79,80]; 3) educational summaries of expert reasoning [56,57,68,71,76,79,80]. and 4) facilitated discussions between learners and educators [48,71,72,75].

The number of vignettes and associated questions varied across studies. Some studies used single-question vignettes [48,55,71], while others included several questions per vignette, with an average of nine questions per vignette [36,68,70]. Most of the LbC training programs were delivered asynchronously, allowing learners and experts to participate at their convenience [68,70,72,79,80]. However, some programs have adopted a hybrid approach, combining asynchronous self-paced learning with synchronous group discussions to enhance interactivity and real-time knowledge sharing [52,71,72].

### Contexts of LbC training

Regarding the ‘context’ category of the PCC framework, the selected studies show that LbC has been implemented in various training contexts for the education of healthcare professionals. These contexts included undergraduate academic programs [46,48,49,67,69,72,76], continuing professional development [36,50,53,55,71], and clinical training environments [47,68,71,75,79].

### Content analysis

Content analysis revealed five recurring elements considered important for the success of the LbC approach, which are presented here in descending order of frequency of occurrence: 1) Design of vignettes and questions; 2) Involvement of experts; 3) Feedback procedures; 4) Training preparation; 5) Collaboration between designers and experts. For vignettes and questions, studies emphasized the importance of using realistic clinical scenarios that incorporate uncertainty to promote decision-making skills [31,47,49,52,53,54,68,71,75,76,79,80]. The participation of experts, i.e., individuals with relevant expertise in the specific field under study, was deemed essential, as they play a central role in validating vignettes by ensuring content validity content, relevance to clinical practice, and appropriate level of difficulty [36,47,53,68,71,72,75,76,79,80]. The experts’ answers served as a reference for learners, helping them refine their CR and decision-making processes. Feedback was considered paramount in this approach, as it includes expert justifications, statistical distributions of responses, and educational summaries, which reinforce learning by allowing learners to compare their decisions with those of experts [48,49,68,71,72,75,76,79,80]. Careful preparation was also considered essential, including defining the target learners, clarifying the objectives, and ensuring relevance to clinical practice to maximize its impact [31,50,68,71,75,76,80]. As for collaboration between designers and experts, it facilitated the training of the experts involved and promoted exchanges between these actors in order to ensure the quality of the training program, in particular the consistency of the vignettes and the answers provided to the questions. This partnership was considered essential to optimize the relevance and educational value of the training [53,68,71,75,76,79,80].

## Discussion

### Scope and Nature of LbC Research

This review has achieved its objective, which was to describe the scope of studies devoted to the LbC approach for developing the expertise of healthcare professionals. The findings indicate that despite growing interest in LbC, the literature on this approach remains relatively limited and heterogeneous. This reality reflects the innovative and emerging nature of the LbC approach, which has been used with different types of learners, in various disciplines and in different contexts [66,68,70,78,79,80]. The main features of this approach in terms of vignette format, questions, learners’ answers to questions, expert responses, and feedback based on these responses, are followed in all included studies. However, there is some heterogeneity, particularly in how this type of training is developed and how learners receive feedback in the studies included [36,37,46,47,48,49,50,51,52,67,68,69,70,71,72,75,76,79,80]. This study also identified key elements that appear to be important for promoting successful learning through LbC. It was also found that the literature on LbC has some gaps, particularly regarding the methodological design of studies of this approach, the standardization of the process of developing and implementing the approach, and the limitations of assessing the effects and impacts of the approach in the studies found.

### Methodological Considerations in LbC Research

Most studies on LbC rely on cohort-based follow-up designs to explore learner engagement and implementation strategies [68,71,72,79]. Although these studies have consistently reported positive perceptions from learners [67,68,69,72,75,76,79,80], their design does not allow for the determination of the added value of the LbC approach compared to other approaches, such as problem-based learning or case-based discussions [62,63]. Indeed, no study has compared the LbC approach to other educational approaches.

Studies reported that students found the approach engaging, interactive, and beneficial for structuring their clinical reasoning [36,47,48,67,68,69,72,75,76,79,80]. The asynchronous digital format was particularly valued for its flexibility, allowing learners to progress at their own pace [68,70,72,79,80]. This adaptability makes LbC accessible to a broader range of learners, including those in geographically remote areas. The evaluation of LbC training has largely relied on learner satisfaction surveys and self-reported perceptions [67,68,69,71,72,75,76,79,80], a common limitation in health professions education research [58,59].

Furthermore, current studies on LbC lack outcome measures that objectively confirm the expertise skills developed through this approach. For example, it would be relevant to conduct longitudinal studies assessing the contribution of LbC to CR and decision-making over time [68,71,77]. Further studies will need to be conducted to describe a reference framework for the indicators of success of the LbC approach that should be evaluated. In addition, theoretical models of learning in LbC should be refined to better understand the mechanisms by which the LbC approach promotes the development of professional reasoning and decision-making.

### Constituent elements of the LbC approach

This review revealed general agreement on the fundamental methodological elements of LbC training, in line with the published guidelines on its design and implementation [34]. These findings suggest that LbC is adaptable to various disciplines (e.g., medicine, nursing, pharmacy, veterinary medicine, dentistry, speech therapy, and patient partnership initiatives) [67,69,70,72,73,75,76,80], to various areas of competence (e.g., clinical reasoning, professionalism), and learner levels (students and professionals). However, the lack of standardized frameworks for developing vignettes, selecting experts, and feedback procedures highlights the need to develop more detailed methodological guidelines on these aspects of the approach [68,78].

### Key elements for optimizing the LbC approach

The review highlighted five elements to consider to optimize learning through the LbC approach. The first element relates to the realism and authenticity of the vignettes, often cited as essential to effective learning, ensuring that the scenarios accurately reflect real clinical challenges [31,47,49,52,53,54,68,71,75,76,79,80]. The second element concerns the participation of experienced professionals. This is considered essential, particularly for validating educational material and ensuring that the level of uncertainty in the vignettes corresponds to clinical practice. The third element concerns feedback, which may include the degree of consensus or variability in the experts’ responses. It is interesting to note that this degree of consensus on the experts’ responses provides an indication of the level of difficulty and relevance of the vignettes [36,47,53,68,71,72,75,76,79,80]. At the heart of the LbC approach, learners benefit greatly from structured feedback that includes expert justifications, statistical distributions of responses and educational summaries, all of which play a crucial role in reinforcing learning [48,49,64,65,68,71,72,75,76,79,80]. Regarding the fourth element, which concerns training preparation, the studies emphasized the importance of clearly defining the target learners, aligning the objectives with clinical practice and ensuring good coordination between faculty members and vignette designers [31,50,68,71,75,76,80]. Finally, a fifth element, close collaboration, was identified as a key factor in optimizing LbC training. Effective collaboration facilitates the training of experts and ensures that feedback remains relevant to the learning objectives [53,68,71,75,76,79,80]. These methodological elements reflect current recommendations from research on LbC and provide a basis for the development of more structured guidelines for its implementation.

### Challenges and Limitations of implementing the LbC approach

Despite its advantages, LbC presents challenges related to the wide range of decision-making required to make effective decisions, skills that are difficult to acquire through a single learning approach. The approach encourages learners to focus on individual diagnostic or intervention hypotheses, which does not always align with the holistic, multifactorial decision-making required in clinical practice. Furthermore, while LbC is grounded in script theory, real-world decision-making often involves a combination of pattern recognition and analytical reasoning, which may not be fully developed through LbC training alone.

Another significant challenge is the resource-intensive nature of LbC implementation. The development and validation of vignettes, collection of expert responses, and administration of training require substantial institutional support [68,71,78,79,80]. Establishing standardized procedures for these steps would streamline the adoption of the LbC approach across different educational settings.

### Study Strengths and Limitations

This scoping review was conducted using a rigorous methodology, including systematic searches of multiple databases and adherence to established scoping review guidelines [38,39]. The specificity of the “Learning-by-Concordance” concept facilitated the development of precise search strategies developed in collaboration with a health science librarian.

One limitation of this study is the exclusion of grey literature, meaning that unpublished LbC initiatives may not have been captured. Given educators’ growing interest in LbC, many applications of this approach may exist outside the peer-reviewed literature. Furthermore, the heterogeneity of the included studies—in terms of methodological designs, targeted learning domains, and outcome measures—limits the ability to synthesize the findings comprehensively [68,71,76,79,80].

It is important to consider verifying the effects of educational approaches using the most meaningful impact indicators available. Although it remains difficult to measure the impact of educational interventions on health science learners’ ability to improve patient outcomes, some studies in medical education have explored indirect measures, such as practitioner confidence, clinical decision-making effectiveness, or error reduction [60,61]. Future research could explore whether LbC training influences these intermediate indicators, rather than focusing solely on short-term outcomes associated with the acquisition of knowledge or skills that are not easily transferable to clinical practice.

### Implications for Future Research and Training Practices

This review underscores the versatility of the LbC approach in fostering different forms of expertise in health professions education. LbC has been applied in domains ranging from CR and diagnostic decision-making to professionalism and perceptual judgment. Learner populations also varied across disciplines, including medicine, nursing, pharmacy, veterinary medicine, dentistry, speech therapy, and patient partnership initiatives [67,69,70,72,73,75,76,80].

A major takeaway from this review is the need for clearer methodological frameworks to support the implementation of LbC. The development of standardized guidelines for vignette design, expert validation, and feedback procedures will enhance the reproducibility and scalability of this approach [68,78]. Future research should also focus on exploring the cognitive processes underlying LbC learning, incorporating longitudinal studies to assess its long-term impact on professional practice [68,71,77].

Given the consistent commitment of learners and the adaptability of LbC, the approach should be considered a viable strategy for developing expertise in various health professions.

## Conclusion

LbC training represents a structured approach to promoting the development of expertise in healthcare professions. It is distinguished by the importance it places on decision-making in a context of uncertainty and the integration of feedback based on the responses of people with relevant expertise in the field under study. Although current studies highlight its adaptability to all disciplines and levels of learning, methodological variability in its development and implementation remains a major challenge. Future research should focus on establishing standardized frameworks for vignette design, expert validation, and feedback procedures. Furthermore, investigating the cognitive processes underlying LbC learning and its long-term impact on professional reasoning could provide valuable insights for optimizing its implementation in health professions education.

## Contribution to the advancement of health professions education

This scoping review provides the first comprehensive synthesis of research on LbC in health professions education, offering a structured overview of its applications. By identifying key procedural factors in LbC training, this review contributes to refining its implementation and highlights the need for standardized evaluation frameworks.

Beyond methodological perspectives, this study also informs the design of future LbC-based training programs, ensuring that vignette development, expert involvement, and feedback procedures are structured effectively. By clarifying the gaps in current research, this review serves as a basis for advancing both the study and practical application of LbC in health professions education.

## Additional File

The additional file for this article can be found as follows:

10.5334/pme.1658.s1 Appendices. Appendix 1 to 5.
