# Supplementary material for: Learning-by-Concordance Approach in Health Professions Education: A Scoping Review
Source: Perspect Med Educ. 2025 Jul 4;14(1):387–98. doi: 10.5334/pme.1658 (PMC12227079; doi:10.5334/pme.1658)
Supplement: Appendices. — Appendix 1 to 5. [file pme-14-1-1658-s1.pdf]

Appendix 1: Bibliographic search strategies in the various databases explored

Data base	Search date	Search strategy
**MEDline**	**2025/03/07**	Medline:<br>https://ovidsp.ovid.com/ovidweb.cgi?T=JS&NEWS=N&PAGE=main&SHAREDSEARCHID=Jhu6VZIJpWnlRB4bh2f4328UymvJgqNWB5j8NnqpN8gQMZYn5mBnu7mxR7TZGFh<br>Ovid MEDLINE(R) ALL <1946 to March 07, 2025><br><br>1 (learn* adj2 concordance).ab,kf,kw,ti. 24<br>2 (train* adj2 concordance).ab,kf,kw,ti. 66<br>3 ((judgment* adj3 concordance) or (judgement* adj3 concordance)).ab,kf,kw,ti. 43<br>4 (script concordance adj5 activit*).ab,kf,kw,ti. 3<br>5 (educational adj5 script concordance).ab,kf,kw,ti. 6<br>6 (pedag* adj5 script concordance).ab,kf,kw,ti. 2<br>7 or/1-6 136<br>8 exp Education/ 940895<br>9 exp Students, Health Occupations/ 96014<br>10 Judgment/ 22735<br>11 exp Decision Making/ 246236<br>12 or/8-11 1195356<br>13 concordance.ti,kf. 6858<br>14 (learn* or train* or teach*).ti,kf. 626727<br>15 12 and 13 and 14 69<br>16 7 or 15 187

**EMbase**	**2025/03/07**	Embase:<br>https://ovidsp.ovid.com/ovidweb.cgi?T=JS&NEWS=N&PAGE=main&SHAREDSEARCHID=38qDSRmO8M99RpQmVK0srRwaeA4B8iuwUXuV0rFn1mwPzIv8iG1xFAze0p8s5MBkE<br>Embase <1974 to 2025 March 07><br><br>1 (learn* adj2 concordance).ab,kf,kw,ti. 27<br>2 (train* adj2 concordance).ab,kf,kw,ti. 110<br>3((judgment* adj3 concordance) or (judgement* adj3 concordance)).ab,kf,kw,ti. 56<br>4 (script concordance adj5 activit*).ab,kf,kw,ti. 3<br>5(educational adj5 script concordance).ab,kf,kw,ti. 8<br>6 (pedag* adj5 script concordance).ab,kf,kw,ti.2<br>7 or/1-6 199<br>8 exp education/ 1776008<br>9 exp health student/ 158264<br>10 exp decision making/ 512331<br>11 8 or 9 or 10 2255978<br>12 concordance.ti,kf. 10341<br>13 (learn* or train* or teach*).ti,kf. 719293<br>14 11 and 12 and 13 98<br>15 7 or 14 274<br>16 limit 15 to Embase 133
**Web of science**	**2025/03/07**	Web of Science Core Collection:<br>https://www.webofscience.com/wos/woscc/summary/c4f5ed08-09f7-4c60-ae92-91db49b31386-014e9260e7/relevance/1<br>1: (TS=(learn* NEAR/1 concordance)) OR (TS=(train* NEAR/1 concordance)) OR

		(TS=(judgment NEAR/1 concordance)) OR (TS=(judgement NEAR/1 concordance)) OR (TS=(script concordance NEAR/4 activit*)) OR (TS=(educational NEAR/4 script concordance)) OR (TS=(pedag* NEAR/4 script concordance))<br>Date Run: Mon Mar 10 2025 12:20:36 GMT-0400 (heure d’été de l’Est nord-américain)<br>Results: 112
**Cinhal**	**2025/03/10**	CINAHL:<br>Mon, mars 10, 2025 3 h 51 min 09 s p.m.<br>Results<br>S15<br>(S7 OR S14)<br>85<br>S14<br>(S12 AND S13)<br>41<br>S13<br>((TI concordance OR MW concordance)) AND (((TI learn* OR MW learn*) OR (TI train* OR MW train*) OR (TI teach* OR MW teach*)))<br>72<br>S12<br>(S8 OR S9 OR S10 OR S11)<br>1 227 675<br>S11<br>(MH "Decision Making+")<br>163,129<br>S10<br>(MH Judgment)<br>7,723<br>S9<br>(MH "Students, Health Occupations+")

		102,474<br>S8<br>(MH Education+)<br>1 099 568<br>S7<br>S1 OR S2 OR S3 OR S4 OR S5 OR S6<br>54<br>S6<br>((TI pedag* OR AB pedag* OR MW pedag*) N4 (TI "script concordance" OR AB "script concordance" OR MW "script concordance"))<br>0<br>S5<br>((TI educational OR AB educational OR MW educational) N4 (TI "script concordance" OR AB "script concordance" OR MW "script concordance"))<br>4<br>S4<br>((TI "script concordance" OR AB "script concordance" OR MW "script concordance") N4 (TI activit* OR AB activit* OR MW activit*))<br>1<br>S3<br>(((TI judgment* OR AB judgment* OR MW judgment*) N2 (TI concordance OR AB concordance OR MW concordance)) OR ((TI judgement* OR AB judgement* OR MW judgement*) N2 (TI concordance OR AB concordance OR MW concordance)))<br>18<br>S2<br>((TI train* OR AB train* OR MW train*) N1 (TI concordance OR AB concordance OR MW concordance))<br>23<br>S1<br>((TI learn* OR AB learn* OR MW learn*) N1 (TI concordance OR AB concordance OR MW concordance))

Appendix 2: Characteristics of articles that met the inclusion criteria (presented in chronological order of publication)

First author (year of publication)	Type of article	Study main objective	Study design	Study population	Type of LbC intervention	Study main outcomes measures
Hornos (2013)[50]	Implem [illegible]	To report on the development and initial implementation of an online training program	**Quantitative**	Physicians (1349 pediatricians; 552 cardiologists)	Concordance of reasoning with 240 vignettes (One-year online continuing professional development program)	Feasibility<br><br>Characteristics of enrolled learners<br><br>Dropout rates<br><br>Participant satisfaction

		8	
**Google Scholar**	**2025/03/23**	79	learning; concordance "Learning by concordance"

						(survey)
Foucault (2014)[66]	Implem	To evaluate the use of the Concordance of Judgement Learning Tool (CJLT) to support ethical decision-making in medical students	**Mixed**<br><br>(Survey + Focus groups)	55 clerkship students (University of de Montreal)	Online CJLT presents ethical dilemmas, students compare their decisions with those of a panel of experts, reasons behind the experts' choices are provided	Critical thinking and ethical reasoning among learners, Usability and accessibility of online training
Foucault (2015)[49]	Implem	To present the implementation of a training program based on concordance of judgment to develop professionalism	**Mixed**<br><br>Qualitative: Thematic analysis (focus group)<br><br>Quantitative : Descriptive statistics (survey)	3rd and 4th year medical students (n=79)	Concordance of judgment<br>20 vignettes with 54 questions (Cronbach's alpha of 0.64 for the 54 questions)	Student satisfaction (survey)<br><br>Experiential learning on professionalism (focus group)
Fernandez (2016)[48]	Implem	To investigate whether students feel they are learning from LbC trainings and whether they find the	**Qualitative**<br><br>Thematic analysis (free qualitative comments on surveys)	1st, 2nd and 3rd cycle medical students (n=300, n=241, n=147)	Concordance of reasoning<br>6 vignettes with 24 questions (1st year haematology course)<br><br>Concordance of perception	Qualitative comments from students who took part in training courses (survey)

		courses relevant			10 vignettes with 10 questions<br><br>Concordance of judgment<br>20 vignettes with 20 questions	
Funk (2017)[67]	Implem	Examine the use of a formative script concordance test in a pharmacy course, evaluate the usefulness of training n	**Qualitative**<br><br>Descriptive study, formative assessment using SCT	170 second-year pharmacy students (University of Minnesota)	25 SCT cases on diabetes and metabolic syndrome, expert panel of 20 pharmacists, formative assessment with classroom discussions	Student learning<br><br>Student perceptions of the training program
Charton (2018)[68]	Implem	To develop and evaluate a mixed (reasoning and perception) LbC training program for ECG interpretation in general practice	**Mixed**<br><br>Quantitative: satisfaction and cognitive load survey<br><br>Qualitative: thematic analysis (focus groups)	120 general medicine interns (Strasbourg and Besançon, France); 10 participated in focus groups	Mixed LbC (reasoning and perception): 20 clinical vignettes with ECGs	Student perceptions (realism of vignettes, usefulness, student engagement and reflection)<br><br>Cognitive load perceived by students
Lecours (2018)[36]	Implem	Investigate whether LbC training is	**Mixed**<br><br>Qualitative :	Family doctors (n=45)	Concordance of reasoning<br>8 vignettes with 77	Participant satisfaction (survey)

		relevant to continuing education	Descriptive analysis (focus group)<br><br>Quantitative : Descriptive statistics (survey)	Expert panelists (dermatologists n=4; senior residents n =2)	questions	Comments from participants (focus group)
Tedesco-Schneck (2019)[69]	Implem	To implement and evaluate a Script Concordance Activity combined with a Think-Aloud (SCA-TA) approach to foster clinical reasoning in nursing students	**Qualitative**<br><br>Descriptive implementation study with anonymous post-training survey	52 senior undergraduate nursing students (pediatric course); 45 completed the perception survey	SCA-TA: 6 class sessions comprising 3 script concordance cases per session (total = 18); students responded using a Likert scale and written justification, then participated in a class discussion (TA debriefing); expert responses were used to generate scoring, but were not directly communicated to students	Students' perceptions of the training
Deschênes (2020)[70]	Devel	To present the educational principles and practical steps involved in designing a LbC aimed at	**Descriptive theoretical (no empirical data collection)**<br><br>Description of the principles,	Not applicable (target audience: college educators)	Presentation of LbC principles applied to college-level scenarios; including sample vignette, responses on a Likert-scale, feedback based on the	No empirical data collected; the article proposed a structured design model for LbC, discussed its educational value, and offers examples of classroom

		fostering professional reasoning in college-level education	concepts, and steps associated with designing a LbC activity		justifications of a panel of experts, and structured summary	adaptations; it highlighted the benefits for reasoning under uncertainty and cognitive scaffolding
Deschênes (2020)[47]	Implem	To develop, test and evaluate a digital educational device based on LbC with nursing students.	**Mixed**<br><br>Research and development design<br><br>Qualitative: Descriptive analysis (logbook and discussion group)<br><br>Quantitative : Descriptive statistics (survey)	Nursing students 1st cycle (n=45)<br><br>Expert panelists (n=12)	Concordance of reasoning<br>22 vignettes with 81 questions	Validation of educational content (modified Delphi method)<br><br>Psychometric characteristics of LbC questions<br><br>Acceptability and usability of the training system (questionnaire and focus groups with students and panellists)
Henriksen (2020)[71]	Implem	To implement a LbC activity with multi-source feedback to address emerging clinical questions	**Descriptive implementation report (no formal data collection)**	23 healthcare professionals involved in frontline COVID-19 care; expert panel composed of 7 specialists (internal medicine,	LbC activity based on real clinical cases submitted by practitioners; 4 vignettes created from these questions, validated by a panel of 7 experts; training participants answered	Discussion on the educational benefits and challenges of LbC

		during the COVID-19 pandemic		microbiology, epidemiology, public health, pulmonology)	using Likert scales, received expert justifications, and participated in a 60-min webinar	
Jackson (2020)[72]	Implem	To design and implement an interpretative LbC activity aimed at training learners to recruit patient partners for healthcare projects	**Descriptive implementation report (no formal data collection)**	Healthcare students and professionals enrolled in an online university course on patient partnership (number of participants not specified)	Interpretative LbC : 8 video clips from real recruitment interviews; learners rated the appropriateness of patients' answers using a 4-point Likert scale, based on several criteria derived from a competency framework; feedback included the distribution of expert responses, written justifications, and educational summary	Discussion on stakeholder participation and reactions to the activity (learners and experts)<br>Comments on the educational value of the training
Deschênes (2021) Script[73]	Devel	To describe the theoretical foundation and educational applications of the Script Concordance Approach (SCA) in nursing education	**Descriptive theoretical paper**	Not applicable (conceptual article)	Digital Educational Strategy Based on Script Concordance (DESBSC), an online LbCtool integrating expert feedback and structured reflection for nursing students. Also discusses face-to-face SCA activities and the use of SCT for	The SCA supports clinical reasoning by exposing learners to uncertainty, comparing their judgments to expert responses, and integrating structured feedback. Grounded in script theory and cognitive apprenticeship, it scaffolds learning

					assessment.	through expert modeling. The DESBSC aligns with LbC principles, offering structured expert justifications to guide learning.
Deschênes (2021)[53]	Devel	To describe the key elements that supported the pedagogical design of an LbC tool from the perspectives of educators and practitioners.	**Mixed**<br><br>Descriptive qualitative research design<br><br>Qualitative: Descriptive content analysis (surveys, written comments)<br><br>Quantitative : Descriptive statistics (surveys)	Educators Authors of the vignettes. (n=6)<br><br>Panelists Doctors & residents (n=19)	Concordance of reasoning<br><br>85 vignettes with 272 questions in seven medical disciplines (family medicine, emergency, psychiatry, pediatrics, musculoskeletal, rhumathology, surgery and gyneco-obsterics)	Educators' perspectives<br><br>Panelists' perspectives
Patel 2022[74]	Implem	Explore the learning experiences of general practice trainees using a formative think-aloud Script	**Qualitative**<br><br>Semi-structured interviews and thematic analysis	11 first-year general practice trainees (Oxford, UK)	Think-aloud SCT format: students answered 30 SCT questions and provided written justifications. Their answers were compared to an expert panel, and feedback	Identified three key learning themes: developing complex decision-making skills, self-evaluation and awareness, and strengthening professional identity

		Concordance Test (SCT)			was followed by group discussion and debrief.	
Tayce (2022)[75]	Implem	To develop and implement a modified Script Concordance Test (SCT) to promote clinical reasoning during veterinary clinical rounds	**Descriptive implementation report**<br><br>Analysis of student feedback (informal, voluntary survey)	Veterinary students (4–6 per rotation); 61 feedback surveys collected over 1 year	Modified SCT: 3-point Likert scale + free-text justifications; completed during clinical rounds (4 vignettes per session); expert responses used to generate feedback prompts for group discussion	Positive student perception; enhanced clinical reasoning and group discussion; justifications revealed variation in reasoning despite similar scores; instructors used real-time feedback to guide teaching; well-suited to formative use
Bernard (2023)[76]	Implem	To design and assess a training program integrating Learning-by-Concordance (LbC) and structured reflection to improve early clinical reasoning in speech-language therapy students	**Quantitative**<br><br>Quasi-experimental study comparing two student cohorts (control vs. intervention), using a custom assessment grid and statistical analysis	35 first-year undergraduate students in speech-language therapy (Université de Limoges); 19 in control group (2020), 16 in intervention group (2021)	LbC elements embedded in course structure (vignettes, video-based case studies, expert scripts, Likert-based SCTs); implemented across three first-semester teaching units; training included 4 structured case discussions with alternating hypothesis generation and expert-supported reflection	Students in the intervention group showed significantly better results in 5 of 9 reasoning indicators (data extraction, semantic transformation, hypothesis generation, orthophonic analysis); tool and method considered promising for early script construction and clinical reasoning development
Lafond	Implem	To measure	**Mixed**	2nd year	Concordance of	Student perception

(2023)[37]		student perception of learning-by-concordance of perception tool's usefulness and ease of use.	Qualitative : Qualitative analyse (comments provided by students) Quantitative : Descriptive statistics (survey)	medical students (n = 879)	perception<br><br>10 vignettes with 10 questions	about tool utility and ease of use (survey)
Maftoul (2023)[51]	Implem	To report on the experience of practicing LbC with speech-language pathology students	**Mixed**<br><br>Quantitative : Descriptive statistics<br><br>Qualitative: Thematic analysis	Speech-Language pathology students (n=71)	Concordance of reasoning<br>12 vignettes	Degree of concordance between student and panelist responses<br><br>Student appreciation
Fernandez (2023)[54]	Devel	To gain a deeper understanding from experienced LbC designers to better support clinician educators' broader uptake	**Qualitative**<br><br>Dialogic action research approach (Thematic analysis)	8 medical, physiotherapy and nursing educators and 2 researchers, all of whom have had experience with LbC or SCT	Concordance of reasoning<br><br>Concordance of judgement	Opinions of educators experienced in designing LbC (dialogue-group sessions)

		of LbC.				
Charton (2023)[46]	Implem	To document the impact of an ECG LbC training program	**Qualitative**<br><br>Thematic analysis (Focus groups and individual interviews)	Medical students (10 participants in focus groups and 6 in individual interviews)	Concordance of reasoning<br>18 vignettes	Participants' opinions (survey and interview)
Deschenes (2024)[77]	Devel	Validate a clinical reasoning assessment rubric based on script theory for use in Learning-by-Concordance (LbC)	**Modified e-Delphi study**<br><br>2 rounds with expert consensus	17 experts in health education (nursing, physiotherapy, dentistry, speech-language pathology, medicine)	Rubric assessing three cognitive processes in LbC: mental representation, semantic transformation of data, and hypothesis generation/confrontation (not tested with learners in this study, developed through an expert consensus process).	All descriptors across three criteria (mental representation, semantic and syntactic transformation, hypothesis generation/confrontation) reached ≥0.9 in clarity and content validity. Rubric revised with refined descriptors and capacities. Intended for formative assessment and structured feedback in LbC.
Deschenes (2024)[78]	Devel	Explore beliefs and experiences of educators involved in designing a Learning-by-Concordance	**Qualitative**<br><br>Qualitative interpretive study (Interpretive Description), using 4 semi-	14 participants (éducators): 6 designers, 4 panelists, 4 health education researchers (nurses, physicians,	LbC used in initial and continuing education across health disciplines (nursing, pediatrics, public health)	Three main themes: (1) need for peer reassurance, (2) impostor syndrome among educators, (3) concerns over the quality of instructional content. Findings highlight the

		(LbC) tool	structured dialogue groups	physiotherapists, speech therapists, dietitians)		importance of authenticity in conveying clinical reasoning and the challenge of making decisions under uncertainty explicit.
Lorenzo (2024)[55]	Devel	To compare response patterns between two groups of primary care physicians (PCPs) and patients.	**Mixed**<br><br>Quantitative Descriptive and inferential statistics of response patterns.<br>Qualitative Inductive thematic analysis of panelists' justifications	Panelists (70 patients and 40 primary care physicians)	Concordance of judgment<br>15 vignettes (one question per vignette)	Response patterns<br><br>Justifications of panelists
Pietrement (2024)[31]	Devel	To propose a framework for creating a LbC training	**Qualitative**<br><br>Nominal group	Pediatricians and/or specialists in medical education (n=5)	Concordance of reasoning<br><br>In pediatric rheumatology	N/A
Vaillant-Corroy (2024)[52]	Implem	To evaluate the feasibility of the use of concordance of judgment learning tool in	**Mixed**<br><br>Quantitative Descriptive statistics on responses to	Third- and fourth-year Canadian dental students (n=33)<br><br>Fourth- and fifth-	Concordance of judgement<br><br>12 vignettes (Canadian students)	Patterns of participant responses to concordance questions

		the context of dental education	concordance questions and training evaluation questionnaire<br><br>Qualitative Analysis of comments to open-ended questions in the appreciation survey	year French dental students (n=87)	7 vignettes (French students)	Participants' appreciation of the training
Verillaud (2024)[79]	Implem	Assess feasibility and perceived value of LbC for training Otolaryngology-Head and Neck Surgery (OHNS) residents in epistaxis management	**Qualitative**<br><br>Online implementation and resident feedback survey	37 OHNS residents (Île-de-France); 25 completed the LbC activity	Online LbC tool with 6 clinical scenarios on epistaxis; for each, residents rated decisions on a 5-point Likert scale, compared their answers with a 4-member expert panel, accessed expert justifications, and educational summaries with hyperlinks	92% reported improved clinical reasoning, 96% improved knowledge of epistaxis; 88% overall satisfaction; high appreciation for expert diversity and realism; some found the 5-point scale and variability in expert answers challenging
Mainville (2025)[80]	Implem	Introduce a Learning-by-Concordance (LbC) tool for pediatric dental traumatology	**Mixed**<br><br>Descriptive quantitative analysis + survey with	n=121 fourth- and fifth-year dental students from two universities (Canada and	33 clinical vignettes on dental trauma with diagnostic, therapeutic, and follow-up questions; expert panel responses; structured feedback	High student satisfaction (95% found it relevant to training); preference for 3-point scale among novices; feedback and expert justifications

		education and compare different modalities	open comment	France)	(justifications and pedagogical synthesis); delivered on Moodle (3-point scale) and Wooclap (5-point scale)	perceived as helpful; students highlighted educational value and suggested improvements (interactivity, clarity, usability)
**Legend**<br>N/A: Not applicable; Implem = implementation of LbC training; Guide = guidelines; Devel= Development of LbC training						

Appendix 3: Main results of selected articles presenting measures of effects of the implementation of LbC training (presented in chronological order of publication).

First author (year of publication)	Type of article	Main results
Hornos (2013)[50]	Implem	Feasibility results<br>One to two months for recruiting experts<br>Three to four months for writing the vignettes<br>The online platform was very helpful in the development process<br><br>Enrolled learners<br>A total of 1,901 participating physicians<br><br>Dropout rates<br>Around 70% of those enrolled pursued the program<br><br>Participant satisfaction (survey)<br>Over 96% of training participants who responded to the questionnaire said they would recommend the program to their colleagues.
Foucault (2014)[66]	Implem	Student concordance scores<br>Mean concordance score: 64.6/100 (SD = 5.1), with a Cronbach's alpha of 0.64 for 54 items.<br><br>Student satisfaction (survey and focus group)<br>Students reported high satisfaction and perceived relevance of the tool. The activity promoted reflection and critical thinking on professional dilemmas.<br>Students appreciated the comparison with expert reasoning and found the online format flexible and user-friendly. Minor concerns about activity length and initial instructions.
Foucault (2015)[49]	Implem	Student satisfaction (survey)<br>76% of respondents said they enjoyed the training overall, and 87% found it realistic and authentic. 78% found it easy to adapt to this new tool. 64% would like to use it to learn in the future.

		Experiential learning on professionalism (focus group)<br>Focus groups suggest that participants have developed skills related to professionalism thanks to the training. Focus group participants said they found the course a little long, but enjoyable and realistic. They would recommend the course to their peers. The instructions at the beginning of the course could be improved by providing an example. The fact that the course was online was an advantage. The feedback from the experts was very formative for their professional judgment. The activity could be made mandatory for all students.
Fernandez (2016)[48]	Implem	Qualitative comments from students who took part in training courses (survey)<br>Learning by concordance of reasoning (first-year medical students)<br>The tool introduces the uncertainty of clinical practice early on, which is difficult but useful. Encourages critical thinking and reflection.<br>The questions are difficult to grasp without prior exposure to the clinical situations concerned.<br>The absence of a "right answer" is a challenge for first-year students, who are embarrassed or disoriented by the ambiguity of expert answers.<br><br>Learning by concordance of perception (second-year medical students)<br>Students perceived that this tool helps to consolidate knowledge and identify gaps, even if it was difficult to try to identify pathologies that had not yet been covered in their curriculum. The radiological images looked very realistic. They would like to have access to the training after they have completed it, particularly to have access to the instructor's feedback and explanations. This training is perceived as more effective than large lecture. They would have liked to have had more vignettes to practice on and more theory on the subject matter before participating in the activity. Teacher guidance is much appreciated.<br><br>Learning by concordance of judgement (clercks in medicine)<br>The students felt that the cases and professional issues presented were appropriate to their level of experience, were realistic and represented situations they had already encountered during their clerkship. They found that the situations presented provided reflective training that could be transferred to other situations with professional issues they might face in their

		future practice. They found the divergence of expert opinions highly formative. However, the synthesis of the panellists' responses, which enabled them to put the answers into perspective, helped to reframe the whole, and was much appreciated. The training enabled them to realize that there isn't necessarily just one right answer when it comes to making a professional judgment. This training is perceived as having added value compared to a theoretical course. The training tool was perceived as pleasant and motivating.
Funk (2017)[67]	Implem	Student performance<br>Mean SCT score: 66% (range 42–85%) among students who answered all questions (n=59).<br><br>Student perception (survey)<br>80% agreed the SCT had a positive effect on their learning; 86% felt it improved understanding of clinical nuances; 73% found the activity more relevant, and 56% more engaging than usual sessions.<br><br>Expert and teaching team feedback<br>Experts (n=20) and instructors endorsed SCT as useful and realistic. The format stimulated discussion and highlighted variability in expert reasoning. Activity retained as formative.
Charton (2018)[68]	Implem	Formative mixed-type LbC (reasoning and perception)<br>Online ECG interpretation training using double expert panels (cardiologists and GPs), without scoring.<br><br>Participant experience (focus groups and survey)<br>FpC format perceived as realistic, flexible, engaging and authentic. The double feedback was valued, and divergent expert answers stimulated reflection. Participants suggested adding post-training debriefings.<br><br>Learning outcomes<br>High perceived usefulness (mean rating: 3.58/4), strong alignment with clinical practice (3.41/4).

		71% of students reported the activity increased their confidence in ECG interpretation. Reflexivity was deeply fostered, with evidence of contextualized learning transfer and professional identity development.<br><br>Cognitive load<br>Cognitive load perceived as high or very high by 74% of students (n=119).
Lecours (2018)[36]	Implem	Participant satisfaction (survey)<br>Participants (n=44) expressed strong satisfaction with the clarity of the training objectives, the user-friendliness of the learning tool, and the usefulness of the experts' explanations. The majority found that the training had improved their knowledge of dermatology (87%), and 84% used a desktop computer to access it.<br>The duration of the training was deemed appropriate by 81% of participants, although 19% found it too short.<br><br>Comments from participants (focus group)<br>Participants (n=5) found the training more effective and stimulating than reading a manual, particularly appreciating the narrated vignettes and the opportunity to reflect before submitting their answers.<br>Access to the experts' answers and justifications helped to improve their clinical reasoning, and the flexible format of the training was appreciated, even after it was over.
Tedesco-Schneck (2019)[69]	Implem	Formative SCT activity using a think-aloud (TA) approach during six pediatric nursing classes.<br><br>Student perception (survey)<br>71% of students reported that the activity improved their clinical reasoning skills.<br>Students appreciated the opportunity to justify their answers and reflect on their reasoning process.<br><br>Learning environment<br>The formative, non-graded nature of the activity reduced stress and increased confidence. Discussion with peers and feedback from experts fostered reflection, salience recognition,

		and prioritization.
Deschênes (2020)[47]	Implem	Validation of educational content (modified Delphi method)<br>The rigor of the search led to the retention of 15 vignettes out of 37 (40.5%) with a Content validity index (CVI) ≥ 0.75, and seven others with a CVI ≥ 0.5 were added to ensure representativeness, totaling 22 vignettes and 81 questions.<br><br>Psychometric characteristics of LbC questions<br>Intra-class correlation coefficients showed the low level of agreement among the three groups of participants' answer choices to the 81 questions. The kappa (k) coefficient was 0.433 among panelists (confidence interval [CI]: 0.347-0.531) showing slightly more consensual answers to the questions than third-year students (k: 0.379; CI: 0.301-0.472) and first-year students (k: 0.34; CI: 0.301- 0,472).<br><br>Acceptability and usability of the training system (questionnaire and focus groups with students and panelists)<br>In the pre-test, a maximum time of 45-60 minutes to complete the educational activity was identified, including suggestions for additional tools to enhance the experience.<br>91% of students were satisfied with the user-friendliness of the device, 97% found the authenticity of the vignettes satisfactory, and 83% considered the training relevant to nurses.
Henriksen (2020)[71]	Implem	Formative LbC training with multi-source feedback<br>Development of rapid-response LbC vignettes on emerging COVID-19 clinical dilemmas, used in continuing professional development (CPD) for physicians.<br><br>Participant feedback (informal feedback and web-based discussion)<br>The activity enabled physicians to compare their clinical judgments with specialists and peers, and fostered dialogue within a community of practice.<br>Participants reported that the format helped clarify uncertainty and update their understanding of evolving clinical knowledge.

		Expert panel and implementation<br>Specialists from multiple domains (n=7) provided justifications; participants (n=23) completed LbC cases followed by a webinar for real-time synthesis.<br>Activity supported remote learning under confinement and allowed rapid dissemination of expert-informed knowledge.
Jackson (2020)[72]	Implem	Interpretative Learning-by-Concordance (FpCi)<br>FpC module designed to train future recruiters of patient partners, using video vignettes and Likert-scale judgments on recruitment criteria.<br><br>Expert involvement<br>Expert panel (n=5) provided responses and justifications based on real-life experience in different recruitment settings. Feedback and synthesis were integrated into Moodle.<br><br>Learner engagement (pilot implementation)<br>The activity stimulated rich discussion on online forums and raised awareness about the diversity of interpretation in patient interviews.<br>Experts reported personal benefits from articulating their tacit knowledge into explicit, shareable feedback.<br><br>Pedagogical innovation<br>FpC was adapted into a format fostering critical interpretation rather than hypothesis testing, introducing the notion of “interpretative concordance”.
Patel (2022)[74]	Implem	Formative think-aloud SCT activity with general practitioner (GP) trainees (n=11) using written justifications and expert panel feedback.<br><br>Student perception (interviews)<br>Trainees found the tool engaging and valuable for developing complex decision-making skills, self-awareness, and professional identity.Think-aloud justification enhanced reflection; expert feedback and peer discussion supported deeper learning.<br><br>Learning outcomes

		The SCT format helped manage clinical uncertainty and promoted reflexivity. Trainees felt reassured when aligned with experts, and the absence of a single "correct" answer was seen as realistic.<br>Participants valued the activity's alignment with real-life GP practice and saw it as a meaningful contribution to their training progression.
Tayce (2022)[75]	Implem	Modified formative SCT used during clinical rounds (veterinary students)<br>Adaptation of the SCT using a 3-point Likert scale and free-text justification field to stimulate reasoning and enable real-time feedback.<br><br>Student feedback (n=61 surveys)<br>Highly positive. Students reported that the activity helped them commit to their reasoning, reflect on uncertainty, and clarify their clinical thought process.<br>Participation increased, including among less confident students.<br><br>Instructor observations<br>Free-text justifications revealed differences in reasoning even when answers were similar. The tool supported individualized feedback and promoted discussion, regardless of student confidence.
Bernard (2023)[76]	Implem	Reasoning-based LbC on ECG interpretation (n=402 invited)<br>Online training with 18 vignettes covering 15 cardiovascular diagnoses.<br><br>Participation and completion<br>35.6% of students (n=143) started the training, and 13.7% (n=55) completed it.<br>Mean completion time: 1h37.<br><br>Student satisfaction (survey)<br>100% found the cases realistic and relevant to ECG training.<br>98.2% said the training was useful for future practice; 89.1% felt more confident interpreting ECGs.<br><br>Cognitive load

		74.5% rated the cognitive load as “rather high” to “very high”, suggesting significant mental effort.
Lafond (2023)[37]	Implem	Student perception about tool utility and ease of use (survey)<br>Students from all three cohorts (three different years) perceived the Perceptual Concordance training as useful and easy to use. Analysis of the data confirms the presence of two distinct factors in their responses. The first factor explains 44.5% of the variance, underlining the perceived importance of the tool. The ANOVA results show reliable mean scores for all three cohorts of students, indicating a consistent appreciation of the tool over the years. Student comments highlight a desire for faster integration of the tool into the program, and a general satisfaction with this type of learning. The results show a positive impact of the tool by perception concordance on the engagement of radiology students, and its recognition as a relevant and easy-to-use pedagogical tool.
Maftoul (2023)[51]	Implem	Concordance of student responses with panelists<br>The students' answers were more consistent with those of the panelists after the "training" section.<br><br>Student appreciation<br>The results obtained from the students testify to their appreciation of this new learning method. According to the students' responses to the reflective evaluation, LbC seems to promote perception of the development of clinical reasoning in learners and the spontaneous adoption of a learning stance.
Charton (2023)[46]	Implem	Participants’ opinions (Focus group and individual interviews)<br>They felt the need to train themselves to read ECGs.<br>They demonstrated attentive, critical, exploratory, and iterative thought processes.<br>Some aspects could be improved to foster the process of reflection. In particular, the long duration and cognitive overload may have had negative impacts on learning.<br>Critical interaction was sometimes limited by the difficulty of understanding the panel answer.<br>They expressed their desire to change their thought processes.<br>Participants noted that the program caused them to consider their clinical reasoning processes.

		When they faced issues related to clinical reasoning, they used resources during the training to develop or create new prototypes and clinical scripts.<br>Clinical reasoning strategies were also guided by emotional factors and uncertainty.<br>The participants easily connected their thoughts to their 'selves' both during and after the training program.<br>Results revealed interesting insights into the impact of the identity of the panel members, the absence of a scoring system and the question of uncertainty in ECG reading.
Vaillant-Corroy (2024)[52]	Implem	Patterns of participant responses to concordance questions<br>Mean score of panel members close to 80/100 (79.9/100 for Canadian panel members and 79.4/100 for French panel members) and a lower mean score for students (66.5/100 for Canadian students and 66/100 for French students)<br><br>Participants' appreciation of the training<br>The students thought that the situations were realistic, promoted reflexivity regarding their future healthcare professional identity, and stimulated the discussion with other students regarding professionalism. They also reported that the educational tool was adapted to the teaching of professionalism. The experience was mostly reported as interesting and relevant. A minority of students reported that the training was too long.
Verillaud (2024)[79]	Implem	Formative LbC on epistaxis management (Otolaryngology (ENT) residents, n=37)<br>Online tool using 6 clinical vignettes and expert feedback (n=4 panelists).<br><br>Completion and satisfaction<br>68% of residents completed the training.88% appreciated the method; 92% wished to use it again; 92% felt it improved their clinical reasoning; 96% reported knowledge gains. All students found the scenarios realistic.<br><br>Perceived learning<br>Residents valued the realism, diversity of clinical contexts, and expert justifications. Diverging expert answers stimulated reflection, though some found the 5-point scale complex or discordant opinions confusing.

Mainville (2025)[80]	Implem	Formative LbC on pediatric dental traumatology (n=121 students)<br>LbC activity with 33 vignettes implemented at Université de Montréal (Moodle, 3-point scale) and Université de Strasbourg (Wooclap, 5-point scale).<br><br>Participation and completion<br>83.9% completion at Montréal (n=68/81); 67.5% at Strasbourg (n=54/80).<br>Perception survey completed by 43.8% of participants (n=53).<br><br>Student perception (survey)<br>100% found vignettes realistic and relevant; over 90% reported improved clinical reasoning.<br>Students appreciated expert feedback and synthesis.<br>Strasbourg students (novices to LbC) found the 5-point scale more difficult to use.<br><br>Learning outcomes<br>The tool helped bridge theory and practice and was perceived as highly relevant, regardless of platform.
**Legend**<br>N/A: Not applicable; Implem = implementation of LbC training; Guide = guidelines; Devel= Development of LbC training		

**Appendix 4:** Main results of selected articles presenting studies on the development of LbC training (presented in chronological order of publication)		
**First author (year of publication)**	**Type of article**	**Main results**
Deschênes (2020)[70]	Devel	Pedagogical rationale<br>FpC is presented as a cognitive apprenticeship strategy to support reasoning in uncertainty. The article details theoretical foundations, construction steps, and implementation modalities adapted to college-level teaching.<br><br>Scenario design<br>Scenarios are short, ambiguous, and incomplete to reflect real decision-making conditions. Each option is challenged by new contextual data, prompting micro-judgments rated on Likert scales.<br><br>Expert panel use<br>Experts provide answers and written justifications without peer consultation. Feedback is delivered via digital platforms with synthesized messages and educational resources.<br><br>Instructional integration<br>FpC is flexible: usable in-class or online, individually or in groups, and designed to stimulate reflection through expert feedback and peer discussion. Emphasis is placed on formative assessment and contextualized learning.
Deschênes (2021)[73]	Devel	Development of an educational approach integrating script concordance to prepare nursing students for clinical uncertainty.<br>The approach includes:<br>1. Script Concordance Testing (SCT) – A quantitative tool to assess clinical reasoning.<br>2. Face-to-face Concordance Activities – Peer discussions on ambiguous cases.

		3. Digital Learning-by-Concordance (LbC) Platform – Online reasoning tasks with expert feedback.<br>Students compared their reasoning to experts, receiving immediate feedback on decision-making under uncertainty.<br><br>Findings suggest that expert justifications help structure clinical judgment and improve self-assessment of reasoning skills.
Deschênes (2021)[53]	Devel	Educators' perspectives<br>Of the 52 collaborators involved, 25 (48%) took part in the study, the majority aged between 31 and 50 (56%), with over half having less than 10 years' teaching experience (52%).<br><br>Educators mainly used academic sources (50% always, 33.33% almost always), frequent or high-stakes situations (66.67% always, 33.33% almost always), and theoretical knowledge (66.67% always, 33.33% almost always) to develop the vignettes.<br><br>Panelists' perspectives<br>Collaboration between educators and panelists in the design of concordance training is seen as an important way of improving the quality of the tool and the experience of the experts.<br><br>Panelists found the vignettes often relevant, reflecting frequent and uncertain questions of clinical practice, but noted difficulties in justifying answers in uncertain contexts.<br><br>Panelists indicated that the time required to answer 10-15 vignettes (30-50 questions) was less than 30 minutes for 21% of them, between 30 and 90 minutes for 58%, and over 90 minutes for 21%.
Fernandez	Devel	Opinions of educators experienced in designing LbC (dialogue-group sessions)

(2023)[54]		Three general challenges inherent in LbC design that are specific to this type of learning strategy:<br>1-The distinction between pedagogical intent and learning outcome.<br>Participants discussed the challenges of distinguishing between pedagogical intent and learning outcome in LbC clinical case design, emphasizing that cases with "gray areas" are best for learning.<br><br>2- The contextual cues used to stimulate students and advance their learning.<br>Designers use contextual cues from their experiment to challenge students,but encounter difficulties in selecting appropriate additional data to match the initial hypothesis.<br><br>3- The integration of experiential and formalized knowledge for cognitive learning.<br>The integration of experiential knowledge with formalized knowledge is crucial for cognitive learning, and LbC cases are often designed in groups to enrich the contextual aspects.
Deschenes (2024)[77]	Devel	Validation of a clinical reasoning assessment rubric for LbC tools using a two-round e-Delphi study (n = 17 experts from nursing, physiotherapy, medicine, and dentistry).<br>The rubric included three script theory–based criteria:<br>1. mental representation,<br>2. semantic and syntactic data transformation,<br>3. generation and confrontation of hypotheses.<br>Each criterion had three performance levels (insufficient, developing, manifest).<br>A total of 14 descriptors were revised and 6 added between rounds. Consensus was reached on all descriptors (CVI and clarity index > 0.9). The rubric is intended for formative assessment and feedback on written or oral justifications in LbC activities.
Deschenes (2024)[78]	Devel	Qualitative interpretative study exploring the beliefs and experiences of 14 educators (designers and panelists) involved in LbC design across disciplines.

		Three themes emerged:<br>1. Need for peer validation – insecurity and discomfort expressing reasoning in uncertain situations.<br>2. Impostor syndrome – self-doubt about expertise and pedagogical relevance of responses.<br>3. Tension between formal and experiential knowledge – balancing clinical authenticity with educational coherence.<br>Participants valued dialogic design processes and called for clearer pedagogical intent and faculty development to normalize uncertainty in LbC design.
Lorenzo (2024)[55]	Devel	Response patterns<br>There was a significant difference between the responses of the PCPs and those of the patients for nine of the fifteen concordance of judgment questions (60%)<br>Justifications of panelists<br>Although responses were statistically different for nine questions, qualitative analysis revealed common justifications among patients and doctors. Positions were rarely defined by group membership (patient or doctor). Similarly, the main themes invoked to justify a particular position were often shared by both groups. However, the sub-themes varied, and some were more frequently mentioned in one group than in the other. Patients have higher expectations of doctors' professionalism.
Pietrement (2024)[31]	Guide	Summary of operations leading to the creation of a LbC training<br>1- List the problems to be addressed during the LbC training (identification of "problems", scripts).<br>2 -Determine the attributes and values of the chosen scripts.<br>3- Determine the weight of values within their script<br>4- Determine which values the designer specifically wishes to emphasize<br>5- Determine hypotheses related to these attributes and their values<br>6- Construct a small clinical vignette idea that corresponds to the actual practice from which these hypotheses will be derived.<br>7- The final structure combines script - attributes - values
**Legend**		

N/A: Not applicable; Implem = implementation of LbC training; Guide = guidelines; Devel= Development of LbC training

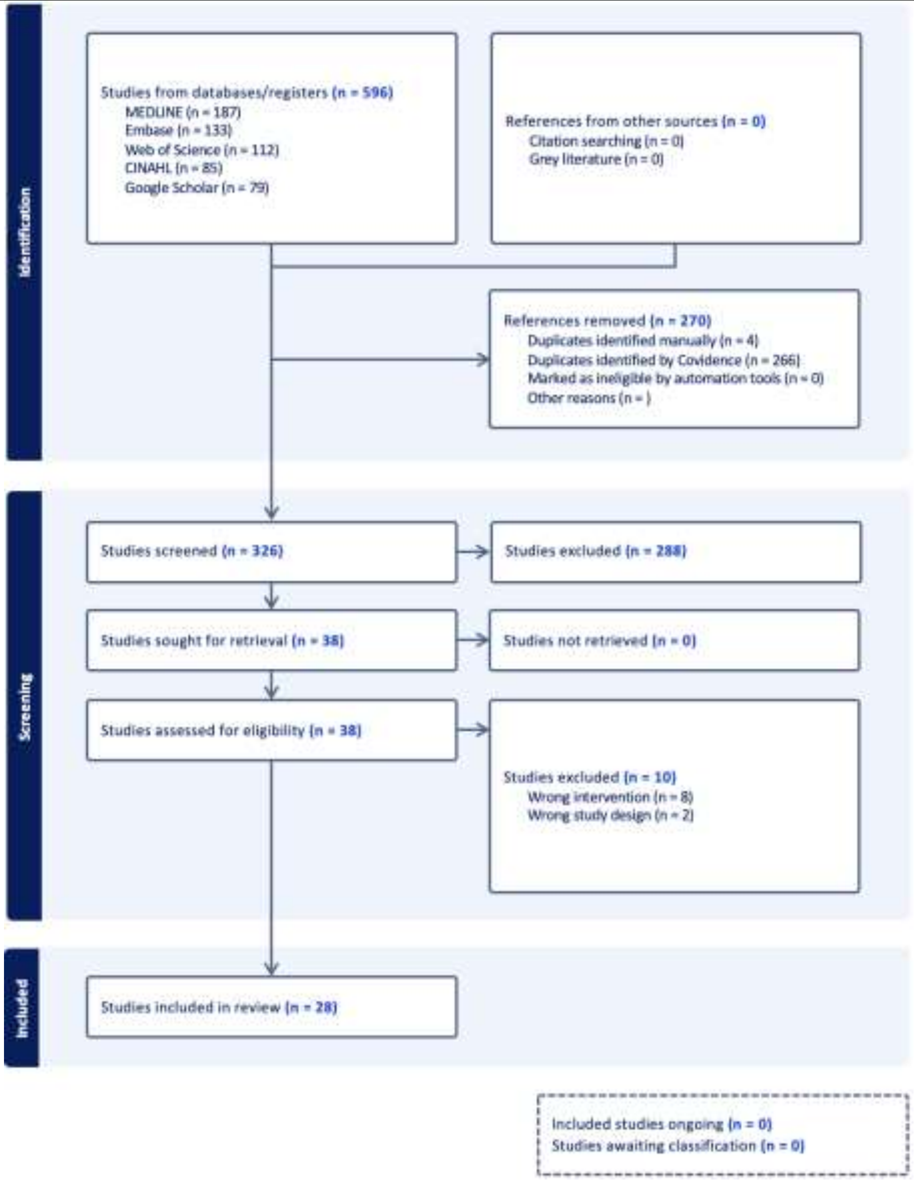

Appendix 5: PRISMA flowchart of the selection process for references included in the review
